# Editorial: Bio A.I. - from embodied cognition to enactive robotics

**DOI:** 10.3389/fnbot.2023.1301993

**Published:** 2023-11-14

**Authors:** Adam Safron, Inês Hipólito, Andy Clark

**Affiliations:** ^1^Center for Psychedelic and Consciousness Research, Department of Psychiatry & Behavioral Sciences, Johns Hopkins University School of Medicine, Baltimore, MD, United States; ^2^Institute for Advanced Consciousness Studies, Santa Monica, CA, United States; ^3^Cognitive Science Program, Indiana University, Bloomington, IN, United States; ^4^Kinsey Institute, Indiana University, Bloomington, IN, United States; ^5^Department of Philosophy, Macquarie University, Sydney, NSW, Australia; ^6^Department of Philosophy and Department of Informatics, University of Sussex, Brighton, United Kingdom

**Keywords:** dynamical systems, e-cognition, embodiment, enactivism, active learning, representation, robotics, artificial general intelligence

## Introduction

“The Brain—is wider than the Sky—

For—put them side by side—

The one the other will contain

With ease—and You—beside—

The Brain is deeper than the sea—

For—hold them—Blue to Blue—

The one the other will absorb—

As Sponges—Buckets—do—

The Brain is just the weight of God—

For—Heft them—Pound for Pound—

And they will differ—if they do—

As Syllable from Sound—”

-Emily Dickinson

If the connections of the human brain were disentangled and placed into a sequence, they would indeed be wider than the sky, being hundreds of kilometers long and likely capable of stretching to the moon and back. If we consider the kinds of intelligence generated by brain-body-environment systems, then such emergent minds may be vaster still in terms of their complex combinatorics, with the pinnacle of expressive power potentially being found in language with its “infinite use of finite means”. The field of artificial intelligence and machine learning (AI/ML) seeks to reproduce the powers of biological learners, where we struggle to recapitulate the ways in which even supposedly simple animals demonstrate the ability to respond flexibly to a wide range of situations. In this Research Topic, we were grateful to receive a diverse assortment of articles that address ways in which principles of enactivism and embodied cognition might allow for advances in AI/ML, potentially without requiring explicit representations, pre-specified algorithms, or centralized control structures. In what follows, we briefly summarize these contributions, highlight some potential implications, and end with a discussion of potential ways forward for AI/ML and cognitive science more generally.

## Summary of contributions and commentary

*Please note that while we use the author's own words where possible, we strongly encourage interested readers to reference the original articles*.

In “*The acquisition of culturally patterned attention styles under active inference*”, Constant et al. present simulations of visual foraging based on active inference, demonstrating the acquisition of attention styles patterned according to cultural artifacts that drive perception, action, and learning. This paper compellingly shows how material culture may both drive and be driven by human thought and by the building and rebuilding of patterns of attention.

In “Enacting plant-inspired robotics”, Lee and Calvo suggest plants as a holistic source of inspiration for soft robotics in terms of their non-centralized, modular architectures and highly plastic phenotypes. In contrast with notions of autonomy based on the independent operability of systems over an observation window, plants and other living organisms exhibit a stronger form of autonomous functioning in terms of needing to support self-production dynamics that create distinctions between themselves and the “domain of interactions that maintain the conditions of viability for the system”. They further suggest that the field of “growbots” could be advanced if those systems took a more active role in acquiring sources of matter and energy for the sake of self-preservation.

In “*Carving up participation: sense-making and sociomorphing for artificial minds*”, Zebrowski and McGraw argue that properly understanding social cognition requires a greater appreciation of the nature of interactions involving participatory sense-making (PSM). “Sociomorphing” is proposed as a means of distinguishing between living sense-makers and artificial systems, potentially allowing for the gradual incorporation of AIs into contexts involving initially asymmetric degrees of sociality. PSM and sociomorphing are suggested to provide not only a basis for social robotics but also a potentially robust framework for developing increasingly advanced AIs with general intelligence.

In “*Embodied object representation learning and recognition*”, Van de Maele et al. show how robotics can be informed by considering the ways in which biological agents achieve scene understanding for adaptive object manipulation and navigation capabilities by leveraging active interactions with the world from their first encounters with novel situations. Taking inspiration from theories of neuroscience in which neocortical columns build predictive models about objects within allocentric reference frames, the authors introduce a Cortical Column Network (CCN) architecture. In CCNs, each object category is represented in its own reference frame by learning a generative model over expected/predicted transformations in pixel space, given actions. CCN ensembles vote on their respective beliefs regarding candidate object categories, which results in the creation of novel CCNs when classification likelihoods are too low. This architecture is further validated in simulation environments, with classification improving as agents gather more evidence (with self-supervised active learning) and choose actions in ways that afford reaching preferred observations/destinations.

In “Grounding context in embodied cognitive robotics”, Valenzo et al. describe how autonomous machines may be augmented with greater behavioral flexibility by providing systems with a “global context” that integrates agent-related, environmental, and task-related information. Through the interaction of these core elements, agents are capable of (1) selecting self-relevant tasks on the basis of current and anticipated future needs (for learning and mastering contingencies), (2) performing tasks with continuous performance monitoring, and (3) abandoning unsuccessful tasks based on overall prediction errors during situated action cycles. With respect to prediction-error monitoring, the rate of reduction is taken as an index of overall performance success, evoking emotions that both function as driving elements for autonomous behavior and are also shaped by the interactions of core elements of global context processing.

In “*The problem of meaning: the free energy principle and artificial agency*”, Kiverstein et al. describe how biological agents solve the “problem of meaning”, by acting in ways that express sensitivity to context-dependent relevance. Drawing on common principles of mind-life continuity and enactivist cognitive science, the authors argue that robustly autonomous agents require stable, self-sustaining patterns of sensorimotor interaction to ground values, norms, and goals as they encounter different (and differently) meaningful environments. The authors further discuss relationships between enactivism and the FEP, including the challenge that these perspectives are fundamentally incompatible, with biological systems exhibiting historical path-dependent learning but with free-energy-minimizing agents severing this historicity. Such FEP agents also show a lack of the “interactional asymmetry” present in enactivist accounts of autonomy. In addition to addressing these challenges, it is suggested that rather than fundamental incompatibility, the FEP needs enactivism for the problem of meaning, and enactivism needs the FEP for precise formal modeling of the necessary constituent factors for realizing agency.

In “*Avoiding catastrophe: active dendrites enable multi-task learning in dynamic environments*”, Iyer et al. introduce a neural network architecture for enhancing the embodied systems to operate in dynamic environments while flexibly adapting to changing task contexts and continuously learning without catastrophic forgetting/interference. This is achieved by incorporating active dendrites and sparsity-promoting local inhibitory systems, so dynamically constraining and routing information in a context-specific manner. The architecture is tested on several benchmarks, including a multi-task reinforcement learning environment in which agents must solve a variety of manipulation tasks (cf. meta-learning), in addition to a continual learning setup in which task predictions change over the course of training (cf. reversal learning). In both simulations, the architecture developed overlapping yet distinct sparse subnetworks that mediated the fluid adaptation to multiple tasks with minimal forgetting, providing (for the first time) a demonstration of high performance with respect to both multitasking and continual learning.

In “*Social neuroAI: social interaction as the “dark matter” of AI*”, Bolotta and Dumas introduce a three-axis framework for social learning in biologically-inspired AI, informed by FEP-AI: (1) brain-inspired models of cognitive architectures, such as global workspace and attention schema theories, that bridge individual and social intelligence; (2) dynamical systems perspectives for handling the inherently time-dependent nature of cognition; (3) embodiment as a source of sophisticated communicative signals. These social interactions are essential elements of advanced cognitive ability yet remain under-explored in AI, constituting the “dark matter” with respect to attempts to understand human(imal)-like intelligence. In light of this gap in our understanding, the authors review the role of social learning in cognitive development and the emerging field of “Social NeuroAI.”

In “*Goal-oriented behavior with a habit-based adaptive sensorimotor map network*”, Woolford and Egbert present a habit-based robot controller model that draws on enactivist principles to realize agency via an adaptive sensorimotor map (ASM) network architecture. ASM networks provide platforms for experimental investigation that combine (1) mechanisms for generating continuous motor activity as a function of historical trajectories and (2) evaluative mechanisms that reinforce or weaken those trajectories as a function of their support for the structure of higher-order sensorimotor coordination. The authors deploy these adaptive networks in a minimal cognition task involving object discrimination, demonstrating how an individual robot could learn through a combination of exploratory/random movements and repetition of successful historical trajectories of sensorimotor coordination (cf. motor babbling). These robots display learning without explicit representational mechanisms or extraneous fitness variables but rather adapt according to the internal requirements of the action-generating mechanisms themselves.

In “*Embodied intelligence: smooth coping in the learning intelligent decision agent cognitive architecture*”, Kronsted et al. describe how skillful actions may become habituated and ingrained through experience, thereby placing less stress on cognitive load relative to considered and deliberative thought and action (e.g., walking, driving, skiing, playing music, short-order cooking). Smooth coping behaviors appear to be automatized in that they are rapid and lacking in reflection, corresponding to Hurbert Dreyfus' description of Heideggerian phenomenology involving “mindless” absorption in action and being in a state of flow. However, pragmatists such as John Dewey et al. suggest that intelligent flexibility is built into smooth coping in ways that make it distinct from automatization. The authors detail a conceptual model of smooth coping using the Learning Intelligent Decision Agent (LIDA) system, informed by the Global Workspace Theory of Consciousness, and argue that sequences of automatized actions are intermittently interspersed with skillful and flexible adjustment by consciously-mediated action selection (via dorsal stream processes). An Automatized Action Selection sub-module is introduced into LIDA to demonstrate these principles within a hybrid architecture that allows for a synergistic combination of both enactivist couplings and explicit representation for the sake of more skillful conscious control of behavior.

In “*Situated neural representations: solving the problems of content*”, Piccinini argues that situated approaches to mind based on embodiment, embedding, enaction, and affect (with extension not being relevant to their discussion) are deeply intertwined with neural representation, with such a computational approach “[requiring] embodiment, embedding, enaction, and affect at its very core.” Additionally, situatedness is suggested to be necessary to describe the adaptive shaping of computations in ways that (1) construct representations with original semantic content, (2) automatically coordinate neural vehicles with representational content, (3) allow content to be causally efficacious, (4) allow content to be sufficiently determinate to be meaningful/useful to systems, (5) allow representation of distal stimuli, and (6) allow for the possibility of misrepresentation.

In “*An enactivist-inspired mathematical model of cognition*”, Weinstein et al. outline an enactivist-compliant mathematical framework for natural and artificial cognitive systems that do not attribute contentful symbolic representations to agents but instead model nervous systems, bodies, and environments as “an inseparable part of a greater totality”. Sensorimotor systems are considered to be special cases of (potentially labeled) “transition systems” with connections to deterministic automata. Minimal sufficient requirements are also suggested for the property of “sufficiency”, including optimal attunement of an organism to its environment with sufficient history information spaces.

In “*Using enactive robotics to think outside of the problem-solving box: how sensorimotor contingencies constrain the forms of emergent autonomous habits*”, Egbert and Barandiaran suggest that AI ought to take inspiration from the “precarious, self-maintaining organization of living systems”. They demonstrate how robots controlled by an iterative Deformable Sensorimotor Medium can realize the spontaneous emergence of an organized ecology of habits capable of re-enacting adaptive behaviors, with habits formed within modalities having relatively greater similarity to habits across modalities (similar to observations for biological systems). These findings are further discussed in terms of their relevance to sensorimotor contingency theory, adaptationist and structuralist explanations in biology, and the potential limitations of functionalist problem-solving approaches to AI.

In “*Reach space analysis of baseline differential extrinsic plasticity [(DEP)] control*”, Birrell et al. introduce a learning rule studied in the context of goal-free simulated agents that produce environmentally aware behaviors. They further extend this mechanism to intentional behavior to determine whether “short-circuited DEP” can generate desired trajectories in a robotic arm via simple open-loop control, with transient and limit cycle dynamics explored in experiments involving target reaching and circular motions.

In “*Resonance as a design strategy for AI and social robots*”, Lomas et al. explore the relationships between the physical mechanisms of resonance and human experience, with consideration for enhancing those (potentially highly impactful) experiences within human-robot interactions. The authors discuss resonance as a cultural and scientific metaphor and review “sympathetic resonance” as a physical mechanism (including synchronization and rhythmic entrainment) and “design strategy” for shaping interactions between human and non-human systems.

With “*Self-concern across scales: a biologically inspired direction for embodied artificial intelligence*”, Sims focuses on a foundation for intelligence for all biological systems that reflects the existential task of continued viability. Self-concern is introduced as “a property of a complex system that describes its tendency to bring about states that are compatible with its continued self-maintenance”, and a potential means of recapitulating the power (and principles) of human-like intelligence in artificial systems.

With “*Mind the matter: active matter, soft robotics, and the making of bio-inspired artificial intelligence*”, Harrison et al. argue for limitations in the realizability of cognitive phenomena such as memory, learning, goal-directedness, and decision-making. That is, the authors describe how cognition is deeply intertwined with its materiality and corporeality and suggest that progress in AI may require treating the underlying material, living processes as more than mere “hardware” that can be abstracted over without consideration for the soft, active, and plastic details of the particular mechanistic realizers. In short, “the matter matters for cognitive form and function.” With “multiple realisability 2.0”, materiality enables, mediates, and constrains cognition, with precarious conditions for existence being essential for understanding how autonomous systems value, engage, and interact with their environments with a goal-directedness grounded in existential needs of survival, persistence, and reproduction.

In “*Reclaiming saliency: rhythmic precision-modulated action and perception*”, Anil Meera et al. characterize the nature of visual attention and saliency and how standard accounts based on mutual information between current visual information and estimated causes fail to consider the circular causality linking perception and action (including decisions as to where to sample next, given current beliefs). From this perspective, salience is defined as an active inferential process that relies on the basic principles of uncertainty minimization and rhythmic scheduling and attention: precision control, or the confidence with which beliefs can be updated, given sampled sense data. Alternatively phrased, salience is related to uncertainty minimization, underwriting the selection of future sense data, and attention is related to rhythmic precision modulation. Numerical experiments are provided to demonstrate advantages for state and noise estimation, as well as system identification and action selection for informative path planning.

In “*Embodiment enables non-predictive ways of coping with self-caused sensory stimuli*”, Garner and Egbert demonstrate how sensory attenuation for self- (relative to externally-) caused stimuli can be explained enactively. This is contrasted with classical explanations of these phenomena based on efference copies, wherein motor commands are accompanied by copies of signals that predict the likely sensory consequences of that activity, which are then subtracted from the actual sensory input. Genetic algorithms are used in this work to investigate when non-predictive solutions might be viable, which in the simple systems tested involved modifying paper to shape or avoid self-caused sensory inputs (rather than predicting and filtering them out) and sometimes leveraging these self-caused inputs for greater control, all without the need for an explicit internal model.

In “*Am I (Deep) Blue? Music-making AI and emotional awareness*”, Novelli and Proksch provide a review of the applications of AI to creative and emotional artistic endeavors, focusing on musical composition. The authors suggest limitations of systems rooted in current AIs that lack “thoroughly embodied, interoceptive processes associated with the emotional component of music perception and production”. The authors' review presents attempts to combine the impressive power of modern generative models with more human-like emotional/interoceptive processing.

In “*Connecting the free energy principle with quantum cognition*”, Gunji et al. outline a potential conflict between FEP-AI and quantum cognition. While free energy minimization leads to a Boolean lattice of classical logical propositions, quantum cognition leads to an orthomodular lattice of quantum logical propositions. Excess Bayesian inference is introduced, with binary relations transformed from a distribution of the joint probabilities via rough-set lattice techniques.

In “*Small steps for mankind: modeling the emergence of cumulative culture from joint active inference communication*”, Kastel et al. provide a compelling and testable deep active inference formulation of social behavior and simulations of cumulative culture. Cultural transmission is cast as a bi-directional communication process that induces particular convergences (via generalized synchrony) between the belief states of interlocutors. Social/cultural exchange is further cast as a process of active inference, equipping agents with choices regarding who to engage with as communication partners, thus inducing trade-offs between confirmation of current beliefs and exploration of social environments. Cumulative culture emerges from the dynamics of belief updating, with equilibria manifesting as segregation into groups whose belief systems are actively sustained through selective, uncertainty-minimizing, dyadic exchanges. Finally, the nature(s) of these emergent equilibria crucially depend on the precision-weighting of each individual's generative model of their encultured niches.

## Conclusion

Across these contributions, we can see a broad range of views on what it means for a system to be biologically inspired, many of which are still neglected in machine learning. For example, people are increasingly interested in enhancing large language models with “multimodality” and potential grounding via simulation environments (Driess et al., 2023; Yin et al., 2023). However, approaches that attempt to take on enactivist insights are rare, with business-as-usual oftentimes assuming that we might be able to rely on achieving new emergent capabilities with sufficient scaling (Silver et al., 2021). This is in contrast to what might be suggested from fields such as developmental social robotics, which emphasize the conditions for bootstrapping (and grounding) robust and flexibly generative models of systems that “grasp” an organism's meaningful interactions with the environment (Dreyfus, 2007; Tani, 2016; Kolchinsky and Wolpert, 2018; Linson et al., 2018; Bisk et al., 2020; Safron, 20211; Hipólito et al., 2023).

From a radically embodied perspective, one might argue that the entire field of cognitivist deep learning is on shaky foundations by virtue of needlessly appealing to the literal sense of the mind-machine metaphor, i.e. to minds as literal information processors (van Gelder, 1990; Van Gelder, 1995; Hutto and Hipólito, 2021; Beckmann et al., 2023). In their view, because computation and information processes cannot be found “in the wild” independent of human (scientific) practices, the literal sense of the analogy pushes toward a rudimentary view of natural intelligence (even if operationally useful in some circumstances). However, we believe that a more ecumenical approach may be called for if we relax some of the usual assumptions that accompany these more cognitivist notions, which may perhaps be made more powerful (and flexible) when re-represented in more enactivist terms. For example, one may think of a diverse range of scientific representations for understanding biological intelligence without necessarily endorsing that the target being represented entails the ontological properties of the model (Candadai and Izquierdo, 2020; Constant et al., 2020). These include (but are not limited to) the following models (of representation/modeling-like phenomena):

1. Implicit “representation” and generalized stigmergic auto-encoding of action-perception cycles via distributed attractor dynamics over likely patterns of enaction with information continuously with/offloaded into the environment in an extended mind sense (Clark and Chalmers, 1998; Pfeifer and Bongard, 2006; Heylighen, 2016).
2. Partially disentangled features in shared latent workspaces (Bengio, 2017; Thomas et al., 2017, 2018)—possibly centered in posteromedial and lateral parietal cortices (Safron, 2021a)—potentially describable as reduced-dimension manifolds over which neuronal activity evolves (Ji et al., 2023).
3. Predictive modeling of the likely homeostatic consequences of different system-world states by subcortical structures that ground all cognition in the preconditions for successful life management and reproduction (Damasio, 2012; Safron, 2021b; Solms, 2021), thus coupling the individual to phylogenetic (meta-)learning (Campbell, 2016; Ramstead et al., 2018; Botvinick et al., 2019; Safron, 2019; Wang, 2021).
4. Predictive modeling (and thereby control) of these system-world estimates by value-canalized striatal-cortical loops could be understood as conditioning these percepts/concepts on likely patterns of enaction. At hierarchically lower levels, these could take the form of softly assembled coalitions of forward models (cf., amortization and planning as inference) (Botvinick and Toussaint, 2012; Kaplan and Friston, 2018). At intermediate levels of abstraction, these could take the form of (experienceable) patterns of embodied simulation and the structuring of perception by relevant affordances (Cisek, 2007). At higher levels, these could take the form of (not directly experienceable) patterns of recurrent activity (or reservoirs), whose bifurcations/tensors could flexibly parameterize likely patterns of enaction with capacities for evaluating multiple policies (Tani, 2016).
5. Re-representation of these features in the spatiotemporal trajectories of the hippocampal/entorhinal system (Blouw et al., 2016; Whittington et al., 2020; George et al., 2021; Safron et al., 2021; Bengio et al., 2022; Dumont et al., 2023), so allowing for orchestration of large-scale dynamics by likely state transitions for the overall agentic system through time-space, potentially affording some of the kinds of graphical representations associated with “good-old-fashioned AI” and symbolic cognitive science (Gentner, 2010; Crouse et al., 2020).
6. Local object models (Kosiorek et al., 2019; Van de Maele et al.), which would be consistent with characterizations of cortical columns as types of transformers, or Numenta's “1000 brains theory” (Hawkins, 2021). While it is questionable whether every cortical column entails full allocentric object modeling capabilities (Safron et al., 2021), this may be the case for local “modules” that are capable of achieving sufficient degrees of functional closure with respect to being able to inform and be informed by action-perception cycles on the timescales of their formation (e.g., whisker barrels, but not ocular dominance columns). This is an example of how seemingly cognitivist models of mental phenomena involving “representation” may heavily depend on an understanding of enactivist principles to accurately characterize the specific details of the operation.
7. Re-representation of these features through symbolic/linguistic capacities (which are themselves realized as probable patterns of enaction for partially expressed motor sequences/grammars), thus allowing for cognition to be structured/stabilized/expanded according to the combinatorics of syntactic language with its “infinite use of finite means”. By affording multi-level recursive self-referential self-modeling, an additional set of strange-loop-involving (Hofstadter, 2007) virtual machines is placed on top of “cognitive” hierarchies, thereby expanding “cognitive light cones” to indeed be “wider than the sky.”—For a preliminary discussion, see Friston et al. (2023).

In this non-exhaustive list of methodologies, it may be possible to find an inclusive, potentially synergistic, and scientifically valuable middle ground between seemingly incompatible theories on the understanding of the mind. This effort is illustrated in the diverse articles in this collection, ranging from discussions of the centrality and power of morphological computation to demonstrations of the promise of biologically-inspired neural architectures.

It is worth noting that this more ecumenical stance still requires criticality, as we would also caution against assuming that adding seemingly biological features to a system will necessarily improve its intelligent/adaptive functioning. This cautioning may be especially timely in light of trends in AI/ML that attempt to project future gains in performance based on a combination of apparent “laws” of ability scaling with computation, especially when combined with analogies regarding human brains as “neural networks”. Of course, brains are indeed types of neural networks, but they also have multiple heterogeneous subsystems, which, taken together, create a control architecture for embodied agents embedded in environments in which they pursue valued goals, usually developed (or trained) in the context of intelligently-structured socioemotional learning curricula (Tomasello, 2014; Veissière et al., 2019; Safron, 20211). As such, attempts to reduce the sophistication of cognition to a “master algorithm” are likely doomed to failure.

Moreover, a substantial amount of intelligent functioning may be realizable via the morphological “computation” enabled by intelligently designed body plans and their physical reactive dispositions. Indeed, this kind of “offloading” of computational challenges onto (or into) bodies and environments is precisely what we would expect from predictive processing systems as they attempt to achieve adaptive functioning with maximal efficiency. While “explaining away” prediction errors via dynamics closer to primary modalities requires fewer neuronal transactions than leveraging more complicated models, the energetic savings (of minimizing cybernetic entropy) are even greater still if prediction errors never enter nervous systems in the first place because they have been eliminated via (en)active inference (Ramstead et al., 2019). It follows, we believe, that the most fruitful meta-prior/over-hypothesis for enactivism-informed cognitive science would be that when it comes to trying to understand the sources of biological intelligence, one should begin with observational behavior and how cognition emerges from a system's interaction with its context-sensitive environment.

We are grateful to have had the opportunity to help bring together this collection on the diverse ways in which embodiment and environmental interactions provide foundations for cognition, across multiple scales. While it may still be debated the precise ways in which systems must be embodied in order to realize which degrees (and kinds) of intelligence, we would even go so far as to conclude with the maxim: “no body, never mind.” Or, in the words of the great late poet Mary Oliver: “The spirit likes to dress up like this: ten fingers, ten toes, shoulders, and all the rest··· It could float, of course, but would rather plumb rough matter. Airy and shapeless thing, it needs the metaphor of the body··· it needs the body's world··· to be understood, to be more than pure light that burns where no one is-so it enters us··· lights up the deep and wondrous drownings of the body like a star” (Oliver, 1986).

## Author contributions

AS: Writing—original draft, Writing—review and editing. IH: Writing—original draft, Writing—review and editing. AC: Writing—original draft, Writing—review and editing.
